# An explanatory in vitro study on the effect of copper gluconate on ferrous gluconate absorption in a Caco-2 cell model

**DOI:** 10.1186/s13104-026-07804-8

**Published:** 2026-04-08

**Authors:** Yasmine Belabed, Béatrice Lopez, Julie Escola, Océane Gobert, Charlotte PJ Talbot, Frédéric Carrois

**Affiliations:** 1 Laboratoire Innotech International, Groupe Innothera, 22 Avenue Aristide Briand, Arcueil, 94110 France; 2 Eurosafe, Saint-Grégoire, France

**Keywords:** Bioavailability, Caco-2 cells, Copper, Iron, Iron-deficiency, Apparent permeability

## Abstract

**Objectives:**

Anemia is one of the most prevalent nutritional diseases worldwide, primarily caused by iron deficiency. Poor absorption of dietary iron results from its low bioavailability and interactions with food components. In that context, dietary iron supplementation has been widely used to treat iron deficiency anemia. For many years, there has been growing interest in exploring various types of iron supplementation to improve the efficacy of iron supplements, satisfying iron needs and lowering side effects. This study aimed to generate preliminary data regarding the effect of co-administration of copper on ferrous gluconate intestinal absorption. To address this question, the iron absorption from ferrous gluconate was measured using the Caco-2 cell model, by determining apparent permeability (Papp) of ferrous gluconate with or without presence of copper gluconate. These results are based on a single experiment and should be interpreted accordingly.

**Results:**

Under experimental conditions, a 4.9-fold increase in iron Papp values was seen when ferrous gluconate was incubated with copper gluconate. These preliminary findings suggest that co-administration of ferrous gluconate with copper gluconate may enhance iron absorption and would provide an additional and valuable benefit for treating iron deficiency compared to conventional iron supplements with iron alone.

## Introduction

Iron is an essential element of various metabolic processes in humans, including DNA synthesis, electron transport, and oxygen transport. Unlike other minerals, iron is mainly regulated by absorption at the apical membrane of duodenal enterocytes via the ferrous iron transporter [1, 2], which is modulated by dietary and physiological factors [3]. Despite relatively low daily iron requirements (about 10 mg ingested for1 mg absorbed), iron is often a growth-limiting nutrient in the human diet and is a leading cause of anemia, one of the most prevalent nutritional diseases worldwide [4, 5]. Standard oral iron therapies are typically based on ferrous iron salts such as ferrous gluconate, due to their superior bioavailability compared to ferric iron forms [6–9].

Biological interactions between iron and copper have been previously studied [10]. Like iron, copper is an essential dietary micronutrient in humans for proper cell enzymatic function, and is essential for efficient iron uptake and mobilization in mammals [11–13]. For instance, Han et al. [14] showed that copper supplementation using CuCl_2_ improved iron bioavailability in differentiated Caco-2 cells [14].

In this context, this study aimed to investigate whether the addition of copper gluconate, a commonly used organic form in nutritional supplements, could enhance the absorption and bioavailability of ferrous gluconate. The data presented results from one single experiment, and are therefore intended as preliminary evidence rather than definitive conclusions. Apparent permeability coefficient (Papp) of ferrous gluconate, with or without copper gluconate, was assessed using the Caco-2 cell model, a human colon carcinoma cell line and a well-established in vitro system for studying intestinal iron absorption [15].

## Materials and methods

### Caco-2 cell culture

Human intestinal Caco-2 cells (batch n° 241175020524, ReadyCell, Barcelona, Spain, 65 passages) have been seeded and differentiated for 14 days onto 6.5 mm high throughput screening (HTS) Transwell^®^ inserts with semi-porous (0.4 μm) polyester (polyethylene terephthalate [PET]) membrane (ref CLS3368, Corning Inc., Lowell, MA, USA) loaded into 24-well assay ready plates.

Upon reception, Caco-2 cells were left at ambient temperature for 2 days, then placed in a cell culture incubator at 37 °C to allow the shipping medium to liquify and the removal of the 24-well assay ready plates.

The shipping medium was then replaced with fresh Caco-2 cell culture medium (Dulbecco’s Modified Eagle’s Medium (DMEM) - Low glucose, with 1000 mg/L D-glucose and supplemented with 10% (v/v) fetal bovine serum, 2 mM/L glutamine, 100 U/ml – 100 µg/mL penicillin-streptomycin). Nine hundred (900) µL and 300 µL of 37 °C prewarmed Caco-2 cell medium were added into each basal well and each apical well, respectively, and the cells were placed at 37 °C in a 95% air/ 5% CO_2_ humified atmosphere for 3 days before performing the permeability experiment.

Therefore, Caco-2 cells were used after 21-day differentiation as monolayers when they spontaneously reach a mature enterocyte-like phenotype, using the well-established conventional culture protocol [16, 17]. In this study, Caco-2 cells were used for permeability studies, when the expression of transporters reaches its maximum [17, 18].

### Determination of caco-2 cell monolayer integrity

Prior to the permeability experiment, the Caco-2 cell culture confluency was examined by assessing the transepithelial electrical resistance (TEER) of Caco-2 cell monolayer. Measurement of TEER is believed to be a good indicator of tightness of the junctions between cells [19]. Briefly, the TEER is a non-invasive technique, that measures the impedance between the lumen and basolateral tissue. TEER measurements use a constant direct current applied by two electrodes, one connected with the lumen side and the other one with the basolateral side of the test system. By applying Ohm’s law, it is possible to measure the related cell resistance, which is correlated to the integrity of the differentiated cell monolayer [20].

TEER was measured immediately before the permeability experiments as one replicate per insert. Monolayer integrity was determined at 37 °C using the EVOM™ Auto voltohmmeter (World Precision Instruments (WPI), Sarasota, Florida, USA). TEER is expressed as Ω/cm^2^.

At the end of the Transwell permeability assay, cell monolayer integrity was measured by assessing passive paracellular passage of the commonly used marker, Lucifer Yellow, which is easily detectable. The Lucifer Yellow (LY) assay is recognized as a reliable test for measuring barrier integrity.

Results correspond to a paracellular flux index and are expressed as LY apparent permeability (Papp).

### Transepithelial permeability study

The permeability study was performed as to the method described by Tavelin et al. [19]. An incubation time of 120 min was chosen as it allows for detectable transepithelial transport while preserving monolayer integrity.

Ferrous gluconate was used as a 40 mg/mL solution, prepared in a Hank’s balanced salt solution (HBSS) buffered at pH 7.4. Copper gluconate was added to a part of the ferrous gluconate solution to obtain a final concentration of 0.5 mg/mL. These concentrations correspond to the therapeutic concentrations in the 10-mL vial of Tot’hema^®^.

Ferrous gluconate and copper gluconate solutions were filtered through a 0.22 μm filter (Millex^®^-GP, Millipore) and prewarmed at 37 °C before permeability experiments.

On the day of permeability experiments, the cell layers were refed with fresh control medium and allowed to incubate at 37 °C for 15 min prior to electrophysiological readings. All electrophysiological measurements were made in HBSS buffer.

Two conditions of apical to basolateral transport were tested: (1) ferrous gluconate; (2) ferrous gluconate in presence of copper gluconate. Two incubation durations were assessed at the start of the experiment (t0; *n* = 1) and 2 h later (t120; *n* = 3).

**Fig. 1:**
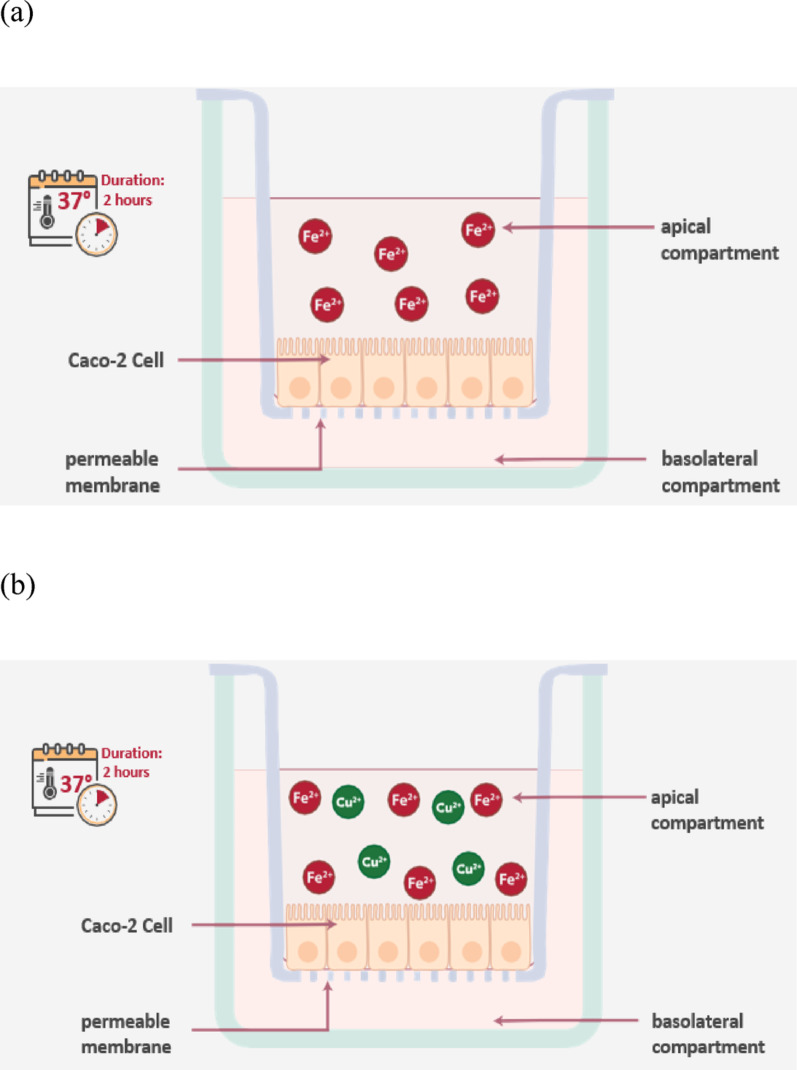
**a** Exposure of Caco-2 cells to ferrous gluconate solution at 40 mg/mL concentration. **b** Exposure of Caco-2 cells to a solution containing a mixture of ferrous gluconate and copper gluconate at 40 and 0.5 mg/mL concentrations, respectively

Iron permeability by mature Caco-2 cells was assayed by loading the apical compartment (250 µL) with ferrous gluconate solution at 40 mg/mL concentration (Fig. 1a) or a solution containing a mixture of ferrous gluconate and copper gluconate at 40 and 0.5 mg/mL concentrations (Fig. 1b), respectively, and the basolateral compartment (750 µL) with HBSS buffer. The plates were placed in a CO_2_ incubator (MCO-15AC, Sanyo, Japan) at 37 °C and the basolateral solutions were collected after 2 h. At the beginning (t0) and at the end of the permeability assay (t120), the entire volume in the basolateral compartment was removed. A part of the initial incubation solutions from the apical compartment was also recovered. All apical and basolateral solutions collected were stored at -80 °C prior to analysis.

### Determination of transepithelial transported iron

Iron concentration was analyzed by inductively coupled plasma optical emission spectrometry (ICP-OES) and the permeability coefficient across the Caco-2 cell monolayers was calculated.

Determination of iron in the specimens collected was carried out on the Thermo Fisher™ ICP-OES iCAP™ PRO system (Thermo Fisher Scientific Inc., Waltham, MA, USA).

### Reagents

Ferrous gluconate hydrate and copper gluconate were provided by Innotech International (Arcueil, France).

Caco-2 cell culture medium (DMEM) and HBSS 1x pH 7.4 buffer were provided by ReadyCell (Barcelona, Spain).

### Data treatment

The intestinal absorption of ferrous gluconate was determined by its apical-to-basal permeability in the Caco-2 monolayer cell model, evaluated with the apparent permeability coefficient (Papp) for iron:

$${\mathrm{Papp~}}={\mathrm{~dQ~}}/{\mathrm{~}}\left( {{\mathrm{dt~x~A~x~C}}0} \right)$$ (1)

where dQ/dt is the cumulative amount of iron present in the basal compartment per unit of time (nmol/s) (Nota Bene: values < 1 have been set to 0 for calculation purposes); A is the transport membrane area of Transwell (cm^2^); and C0 is the initial concentration of iron in the apical compartment (nmol/mL).

### Statistical analysis

Data are presented as mean values ± standard deviation (SD) values of triplicate measures obtained from one single experiment. Statistical analysis was performed using GraphPad Prism (Version 10.4.1, 2024; GraphPad Software, MA, USA). *p* ≤ 0.01 was considered significant and is denoted with two asterisks. Data was analyzed with the Student’s t-test.

## Results

### Caco-2 cell culture integrity

Prior to the transepithelial permeability experiment, the TEER values examined directly before transport were relatively constant, with values ranging between 2125 and 2653 Ω x cm^2^, indicating high integrity of the Caco-2 cell monolayer in the cell culture system [21, 22].

At the end of the permeability assay, the integrity of the Caco-2 monolayer was checked again by examining the paracellular passage of the paracellular marker, Lucifer Yellow (LY).

The LY Papp value was ≤ 1 × 10^− 6^ cm/s, confirming the preservation of a high barrier integrity during the assay [21].

### Transepithelial permeability experiment

After iron analysis of initial incubated samples in the apical compartment, it was observed that iron concentrations measured in the 2 specimens collected (4649 and 4684 mg/L) were in accordance with the theoretical concentration prepared (4792 mg/L).

**Fig. 2:**
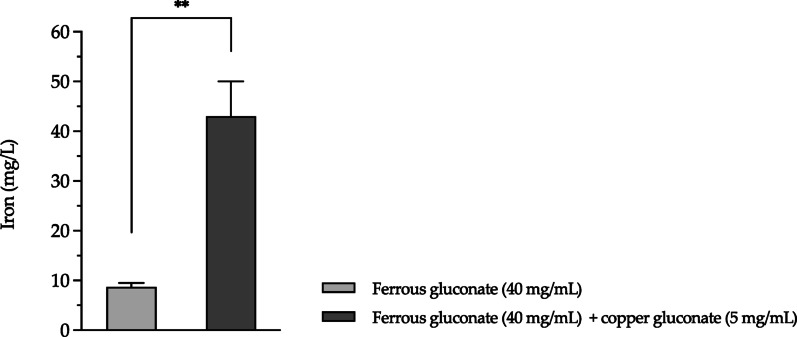
ICP-OES determinations of iron (mg/L) in basolateral compartment, after 120-min incubation, in solutions containing ferrous gluconate or ferrous gluconate and copper gluconate. Data are presented as mean values ± SD of an experiment performed in triplicate. ** p ≤ 0.01

After 120 min of incubation, mean iron concentrations in the basolateral compartment were 8.7 ± 0.82 mg/L and 43 ± 7.0 mg/L for ferrous gluconate and ferrous gluconate/copper gluconate solutions, respectively, both tested at 40 mg/mL in ferrous gluconate (*p* ≤ 0.01) (Fig. 2).

The determination of Papp values in the apical to basolateral side showed that the apparent permeability coefficient of iron was dependent on the presence of copper (Table 1).

**Table 1:** Unidirectional (A → B, from apical to basolateral side) apparent permeability values of iron across Caco-2 cells exposed for 2 h in different solutions of ferrous gluconate

	Ferrous gluconate (40 mg/mL)	Ferrous gluconate (40 mg/mL) + copper gluconate (0.5 mg/mL)	*p*-Value
Mean Papp (x 10^− 5^ cm/s) ± SD	0.059 ± 0.0055	0.29 ± 0.047	≤ 0.01
Papp ratio	4.9		

Data are presented as mean values ± SD of an experiment performed in triplicate.

The results showed a significant 4.9-fold increase in Papp for iron between the copper gluconate-free condition and the condition with copper gluconate, suggesting that the intestinal absorption of ferrous gluconate is increased in presence of copper gluconate (*p* ≤ 0.01).

## Discussion

Results from this study indicate that the transepithelial transport of iron through the Caco-2 cells was increased by a 4.9-factor in the presence of copper, as compared to iron alone, without affecting the integrity of the cell monolayer.

Differentiated Caco-2 cell model is a widely recognized preclinical model for assessing the in vitro bioavailability and safety of different xenobiotics, including iron supplements [23, 24]. Despite some limitations, such as the absence of an outer mucous layer, the absence of the epithelium communication with other organs, and the lack of hepcidin-regulated transport [23, 25–27], it is a relevant model for studying the molecular mechanisms of iron absorption, with good correlation to human bioavailability [14, 25]. Importantly, our findings align with those of Han et al. [14], who observed increased iron transport in differentiated Caco-2 cells under copper repletion. However, their study used longer CuCl₂ exposure and included transporter expression analyses (DMT1, FPN1, hephaestin), whereas our short-term (2 h) co-incubation with copper gluconate assessed only transepithelial iron passage, without evaluating transporter expression [14].

In this study, iron and copper concentrations in the apical compartment of the test system were about 83,000 µM and 1,100 µM, respectively, mimicking the formulation of Tot’hema^®^ but far above duodenal concentrations to be expected after oral administration of Tot’hema^®^. However, the high copper concentrations used in our study did not induce any alterations in the barrier integrity, as indicated by the LY apparent permeability value ≤ 1 × 10^− 6^ cm/s. In addition, the transepithelial transport of iron in the Caco-2 cells was increased by a 4.9-factor in the presence of copper.

The experimental design is limited to a single transport experiment with only technical replicates and no biological replicates, which reduces the robustness of the findings. The observations were only descriptive. Mechanistic aspects of copper-iron interactions such as the expression of key iron transporters and enzymes (DMT1, ferroportin, hephaestin, and ferritin), which could provide insight into the underlying molecular mechanisms, were not assessed. Ferritin, a key intracellular iron-storage protein, is a commonly used surrogate marker of cellular iron handling; its assessment alongside Papp could help distinguish transepithelial passage from cellular retention. The potential competition between Cu²⁺ and Fe²⁺ for shared transport pathways, dose-response effects and potential inhibitory effects at higher concentrations were not addressed either. Only one concentration of copper gluconate (corresponding to the dosage of Tot’hema^®^ formulation) was tested. Finally, longer incubation times may be required to detect changes in surrogate markers of iron absorption, such as ferritin expression, which could strengthen the interpretation of the permeability results.

## Conclusions

This exploratory in vitro study showed that the presence of copper, at the concentration found in the Tot’hema^®^ formulation (0.5 mg/mL), significantly enhanced the apparent permeability of iron from ferrous gluconate by almost five-fold compared to ferrous gluconate alone. These findings suggest that co-administration of iron with copper could improve iron uptake [7, 28–31]. Further investigations with mechanistic studies, multiple doses, and in vivo studies are needed to fully understand and confirm the positive interaction between iron and copper.

## Data Availability

All data generated or analyzed during this study are included in this published article.

## Abbreviations

DMEM: Dulbecco’s Modified Eagle’s Medium
DMT1: Divalent metal transporter 1
DNA: Desoxyribonucleic acid
FPN: Ferroportin
HBSS: Hank’s balanced salt solution
HP: Hephaestin
ICP-OES: Inductively coupled plasma atomic emission spectrometry
LY: Lucifer yellow
MA: Massachusetts
Papp: Apparent permeability
SD: Standard deviation
TEER: Transepithelial electrical resistance
USA: United States of America
